# Long-Term Socioeconomic and Neurologic Outcome for Individuals with Childhood-Onset Multiple Sclerosis

**DOI:** 10.3390/children11081024

**Published:** 2024-08-21

**Authors:** Moritz Tacke, Iris Hannibal, Katharina Vill, Michaela V. Bonfert, Wolfgang Müller-Felber, Astrid Blaschek

**Affiliations:** Department of Pediatrics, Division of Pediatric Neurology, MUC iSPZ Hauner–Munich University Center for Children with Medical and Developmental Complexity, Dr. von Hauner Children’s Hospital, LMU University Hospital, 80337 Munich, Germanyiris.hannibal@med.uni-muenchen.de (I.H.); katharina.vill@med.uni-muenchen.de (K.V.); michaela.bonfert@med.uni-muenchen.de (M.V.B.); wolfgang.mueller-felber@med.uni-muenchen.de (W.M.-F.)

**Keywords:** multiple sclerosis, paediatric onset, early onset, natural history, quality of life, MSIS29, EQ-5D 5L, adolescent medicine, socioeconomic

## Abstract

Intorduction: Most studies on the progression of childhood-onset multiple sclerosis (MS) involve relatively short follow-up periods, focusing primarily on neurological outcomes and disability progression. The influence of these and other factors on the health-related quality of life is not known. To gain a comprehensive understanding of early-onset MS, it is crucial to evaluate the effects of treatment and the disease on quality of life. Method: This pilot project aimed to evaluate the feasibility of using an online survey tool for long-term follow-up data collection from patients with childhood-onset MS. An anonymized, monocentric, prospective survey was conducted on a convenience cohort of patients treated at a certified centre for neuromuscular diseases in childhood between 2007 and 2019. Results: A total of 27 patients completed the survey. There were no mandatory items, therefore some patients chose not to answer all the questions in the questionnaire. Patients exhibited promising educational achievements, low neurological disease burden, and high resilience. However, anxiety, depression, and pain significantly impacted their perceived health status. Conclusion:This single-centre study has yielded new insights into childhood-onset MS. To enable more accurate comparisons across different centres and countries, it is essential to establish a minimum data set and questionnaire subset for patients with paediatric-onset MS transitioning into adulthood.

## 1. Introduction

Multiple sclerosis (MS) is a chronic disease of the central nervous system that typically begins in young adulthood. Patients who develop the disease before the age of 18 account for about 5% to 10% of all MS cases. People with an early diagnosis of MS appear to be particularly vulnerable to the effects of chronic inflammation, although on the positive side there may be better compensatory mechanisms in the brain at a younger age. The higher cognitive reserve may partially mitigate the negative effects of brain damage and may explain the described lower socioeconomic performance in later adult life [1,2].

In principle, the treatment of patients with paediatric-onset multiple sclerosis (POMS) is no different from that of adults. Guidelines exist that define treatment algorithms, even though only a minority of all treatment options are approved for children and adolescents. 

The majority of studies investigating the course of POMS are based on relatively short follow-up periods. They have focused mainly on the neurological symptoms and progression of disability [3,4,5,6]. These are important, but not the only determinants of the health-related quality of life in these patients. Evidence from the literature is sparse and comes from registries in Sweden [1,7] and Italy [2]. 

The comprehensive Swedish registry study encompassed 485 patients diagnosed from 1990 to 2016. The cohort of patients with MS exhibited a lower socioeconomic status compared to the general population. This effect was observed across all age ranges, with a particularly pronounced prevalence among the elderly. The primary objective of the Italian cohort (n = 48) was to examine the cognitive outcome. The presence of cognitive impairment was found to be predictive of a worse socioeconomic outcome. The effect on the quality of life has not been reported.

To better understand the impact of POMS, it is therefore important to quantify the effect of treatment or disease on all domains that may affect quality of life (QoL). As there is no nationwide registry of all patients with POMS in Germany, we are using this pilot project to contact patients who have been treated at our centre between 2007 and 2019. The aim of the project was to get data on the long-term burden of disease in POMS patients and to demonstrate the feasibility and data quality of an online, anonymised survey tool.

## 2. Methods

### 2.1. Study Design

The survey was conducted using an online tool (Lime Survey), which is hosted at the University of Munich. The access code for the Lime Survey was sent to the last documented postal address of the patients. The survey can be found in supplemental data.

### 2.2. Ethics Committee Approval

For data protection reasons, only a completely anonymised survey was permitted (data protection approval dated 10.3.2022, project FG1902). The project was approved by the local ethics committee (22-0844 dated 23 March 2023). 

Due to the completely anonymous nature of the survey, individual consent was not required.

### 2.3. Population and Definition of Cases

Paediatric-onset cases were defined as those with MS onset before the age of 18, according to the definition proposed by the International Paediatric MS Study Group [8]. 

Inclusion criteria: All patients treated at our centre between 2007 and 2019 were contacted, with a minimum age of 20 years chosen to represent a minimum follow-up of 2 years after reaching adulthood.

Exclusion criteria: Patients younger than 20 years at the time of the survey. 

The demographic data requested included the current age and gender. Regarding the progression of MS, patients were asked about the current classification of the disease (relapsing-remitting vs. secondary progressive) and the number and time period of the last relapses. Current complaints/limitations and a current EDSS (Expanded Disability Status Scale) (if known) were requested. The current and past medication was also recorded.

In addition to therapies such as physiotherapy, occupational therapy, or speech therapy, the level of care and the degree of disability were also documented.

The survey asked about school-leaving qualifications, training, and the person’s current occupation and family status.

### 2.4. Questionnaires

The EQ-5D is a patient-reported measure of health status covering 5 dimensions of health (mobility, self-care, usual activities, pain/discomfort and anxiety/depression) and a visual analogue scale (referred to as EQ-VAS). Individuals are asked to report on each health dimension based on the following classification:

- LEVEL 1: indicating no problem
- LEVEL 2: indicating slight problems
- LEVEL 3: indicating moderate problems
- LEVEL 4: indicating severe problems
- LEVEL 5: indicating unable to/extreme problems.

The EQ-VAS is like a psychometer on which people rate their perceived health from 0 (worst imaginable health) to 100 (best imaginable health).

The MSIS-29 is a patient-reported questionnaire that measures the impact of MS based on a set of 29 questions about physical (20 items) and mental health (9 items), with each question having 5 response levels (not at all, a little, moderately, quite a bit, extremely). Cumulative scores are generated separately and transformed to a scale of 0–100, with 0 representing no perceived disability and 100 representing severe disability [9].

Fatigue was assessed using the 6-item general fatigue section of PedsQLFatigue, Version 4.0.

### 2.5. Statistical Analysis

Due to the limited number of respondents in this feasibility study, further statistical analysis of the findings was not attempted. The raw data were analysed using Microsoft Excel. Quantitative values were examined using the sample mean and median as well as the interquartile range (IQR).

## 3. Results

In the period between 2007 and 2019, a total of 60 patients with a confirmed diagnosis of POMS were treated. 12/60 patients could not be contacted because the postal address was no longer correct. A total of 48 patients was successfully contacted, of whom 27 (56%) answered the online survey. Participants were allowed to answer part of the questionnaire, no question was compulsory to complete the survey, so some sections had partial answers.

### 3.1. Demographics/Education

The average age of all patients was 23.7 years (SD ± 3.85), with a minimum age of 20 and a maximum age of 29 years. It was evident from the patient records that only a few of the patients successfully contacted had onset of the disease before the age of twelve (n = 2). The ratio of females to males was 2.8:1, which is to be expected for a predominantly post-pubertal onset of MS. Four participants either did not provide any information or indicated their gender as diverse (Table 1). 

A total of 84% of the patients (21/25) have completed higher education, i.e., more than eight years of schooling. Four patients have completed eight years of education, which is equivalent to lower secondary education. Four patients did not answer this question. 

A total of 15 patients stated that they had already completed vocational training or received a university degree. Of these, 13 patients work full-time and two work part-time on a self-selected basis. None of the patients had to work part-time because of their MS. One in twenty-five respondents said MS prevented them from completing an education. 

A total of 7 of the 24 patients have a disability certificate with a degree of disability between 40 and 70 per cent based on the German scoring system (Supplementary Figure S1). Germany also has special grades, primarily for walking ability or the need for daily assistance. Four participants received a disability mark for walking difficulties. Only one participant was given a disability mark for assistance in everyday life. In addition, only one patient has a care level of 3, which is the middle of five German care levels and indicates “significant impairment of independence”. A total of 25% of patients received physical therapy (6/24), 2/24 received occupational therapy (2/24), 2/24 received psychology/neurocognitive therapy, and none received speech therapy. 

### 3.2. Clinical Characteristics

Five patients did not answer the question in the section on clinical features. At the time of the survey, 22/22 patients reported a relapsing-remitting course. A total of 9/22 patients had a maximum of two relapses since their 18th birthday and only 5 patients had more than two relapses since then. A total of 8 of the 22 patients reported that they had not relapsed since turning 18 years old. Only 1 in 22 patients had a relapse in the previous 2 years. No secondary progressive course was noted. 

The low rate of relapses corresponds to the overall low EDSS as far as known (23/27). Most patients (14/23) had an EDSS of 1, while 7 had an EDSS of 2. Only 2 of the 23 participants had an EDSS of 3 or 4.

**Figure 1:**
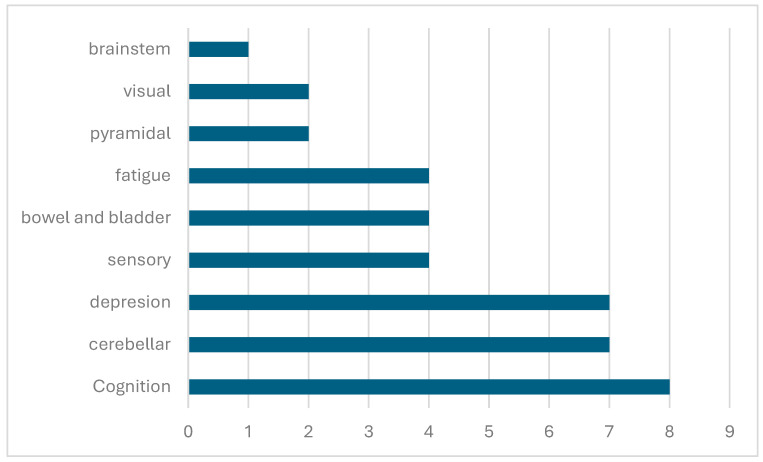
Difficulties in each neurological domain. The absolute number of patients reporting difficulties in each neurological domain is shown.

Participants were asked structured questions about difficulties in various neurological domains (motor, coordination, brainstem, sensory, visual, cognitive, fatigue, and depression). A total of 11/24 patients did not indicate any difficulties, corresponding to an EDSS of 1. Irrespective of relapse, the most commonly reported symptoms in the remaining 13 patients were problems with concentration and memory (n = 8), coordination (n = 7), and depressed mood (n = 7) (Figure 1). Subsequently, four respondents each reported difficulties and restrictions in the sensory system, bowel/bladder, or fatigue. Motor and visual disturbances were much rarer (n = 2 each), and only 1 patient reported brainstem symptoms. The EDSS in this group correspondingly ranged from 1 to 4. Only one patient reported many difficulties in different areas with an EDSS of 1, which did not reflect the actual neurological status expected from the questionnaire. 

**Figure 2:**
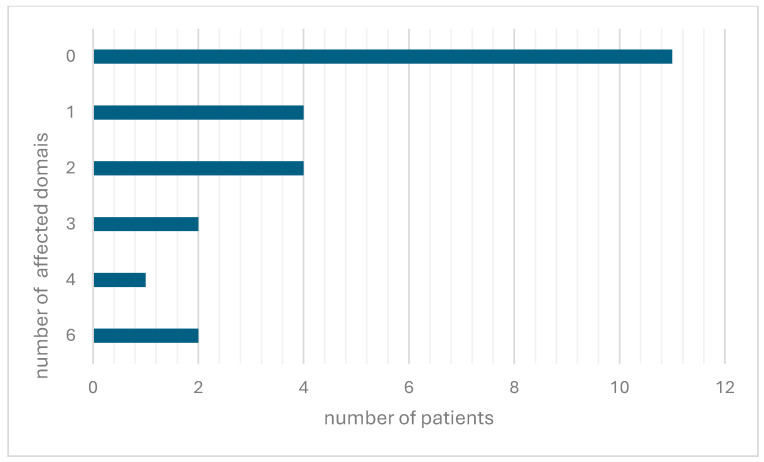
Number of affected domains in patients.

The majority of patients (11/24) report zero affected domains, followed by difficulties in one or two areas. Four or more problems are only described by 3/25 patients (Figure 2). The patient that reported four areas of difficulties (coordination, cognition, fatigue, and depression) had an EDSS of 2. The last known EDSS scores of 1 and 2 were reported by the two patients with six disability areas noted in daily life.

### 3.3. Disease Modifying Therapies (DMTs)

**Figure 3:**
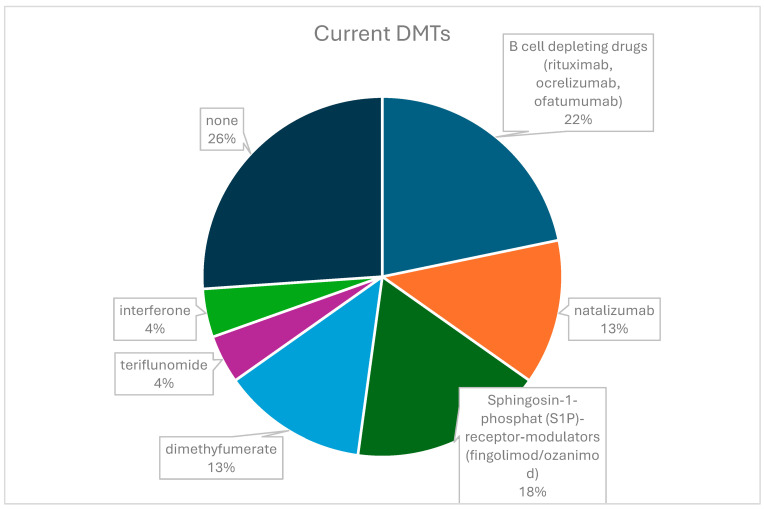
DMTs taken at the time of the survey.

A total of 23/27 patients responded to this section. A total of 17/23 patients were receiving DMTs at the time of the survey, while the remaining patients reported that they were not taking any (Figure 3). The majority of patients receiving DMTs were receiving drugs for highly active MS (70%), with B-cell depleting agents being the most commonly used. A total of 22/23 patients were satisfied with their current treatment. In the past, 18/23 respondents had switched therapies for various reasons. Side effects were by far the most important reason, followed by lack of efficacy (relapse/MRI activity) or a combination of both (Supplementary Table S1).

### 3.4. Quality of Life and Fatigue Questionnaires

#### 3.4.1. EQ-5D 5L

**Figure 4:**
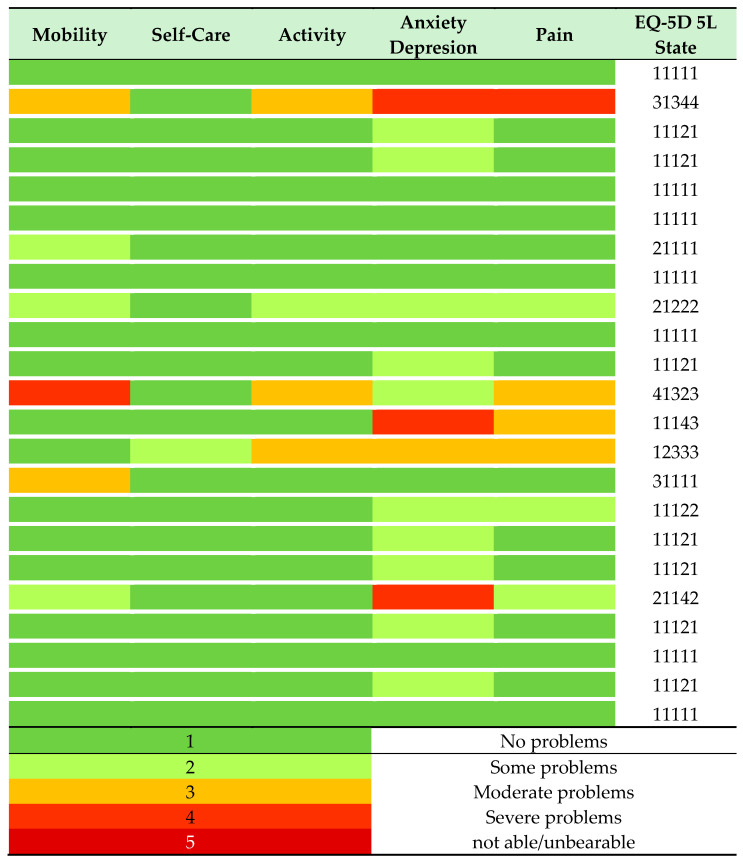
Heat map coding patients self-rated health in each EQ-%D 5L dimension (dark green = no problems, light green = slight problems, orange = moderate problems, red = severe problems, dark red = unable to/extreme). Each row represents the response of a single patient.

The patient-reported measure of health included five dimensions of health (mobility, self-care, usual activities, anxiety/depression, and pain) and a visual analogue scale (called EQ-VAS). A total of 8/23 patients had the best possible health status, with no perceived problems in any of the five dimensions. They are shown in Figure 4 as dark green lines. Ten patients reported at most mild problems in one of the dimensions assessed. Altogether, 18/23 patients had reported their health status to be normal or only mildly affected. The remaining five patients indicated that they had moderate to severe problems with their state of health. It is evident that anxiety/depression and pain have the greatest impact on patients’ perceived health status. The median EQ-VAS (n = 20 respondents) was 90 (IQR 78.75–96.25), with a maximum of 100 and a minimum of 45 being reported individually.

#### 3.4.2. MSIS-29

The MSIS-29 can measure the impact of MS on both physical and mental health. A sum score of physical and psychological health was calculated for each individual patient, with the resulting median MSIS-29 score of 2.03 and an interquartile range of 0–12.5. In contrast, the psychological median MSIS-209 score was higher, with a value of 11.1, while the interquartile range was 2.78–16.67. Details for individual patients can be found in Supplementary Table S2.

### 3.5. Fatigue (6 Item General Fatigue Section of PedsQL)

The answers to the survey questions contribute to an overall general fatigue score. After linear transformation, a higher score indicated less fatigue. A median score of 81.25 was calculated for all patients with an IQR of 63.54 to 91.67. A total of 13/22 (59%) respondents had individual sum scores indicating no or minimal fatigue, corresponding to individual values above 75. A total of 4/22 (18%) reported feeling fatigued sometimes and 5/22 (22.7%) reported feeling fatigued often or almost always. The median, mean, and standard deviation of the PedsQL general fatigue scores for the sample as a whole, as well as for each individual patient, can be found in Supplementary Table S3.

## 4. Discussion

The objective of this study was to investigate the long-term outcome of patients with POMS who were treated at a single centre in Germany. The methodology employed was an anonymised online Lime Survey completed by a total of 27 participants (56%), which is in accordance with the expectations for an online survey conducted without optimisation strategies [10].

### 4.1. Education

All patients had completed their schooling. A majority of 84% (21/25) had completed higher education, i.e., more than eight years of schooling, see Table 2. The published data for all German graduates in 2018 are almost identical, with 82% of graduates leaving school with a higher education qualification [11]. 

A total of 15 patients stated that they had already completed vocational training or a university degree, and only one patient was unable to complete higher vocational training due to MS. Those (15/27) who are already working in their profession have not had to reduce their working hours due to MS. The proportion of those with higher education or unrestricted employment is higher than in a Swedish registry study, in which only 48.5% had higher school education [1]. The Swedish cohort exhibited a greater prevalence of sickness absence or disability payments, which increased with age. The study population differed in that it included patients diagnosed between 1980 and 2014. The majority of patients were diagnosed prior to the advent of highly effective treatment, particularly at the time of diagnosis. The authors describe that stratification of data in an older cohort (onset before 1999) and a younger cohort revealed that the older cohort is primarily responsible for the observed effect. Furthermore, a recent Italian long-term follow-up on POMS patients demonstrated that 21 patients exhibited a mild functional impairment, as measured by the WSAS score [2]. All data combined show a trend toward better socioeconomic performance in patients diagnosed of POMS more recently. 

### 4.2. Clinical Characteristics 

All 22 patients reported a relapsing-remitting course. See Table 3 for the number of relapses. Only one had a relapse in the previous two years. The majority of patients achieved freedom from clinical disease activity through the use of disease-modifying therapies (DMTs) for highly active disease. These included B-cell depleting agents, natalizumab and fingolimod, which were either used as a primary treatment or after switching from another therapy. The shift towards DMTs for highly active disease reflects the change in the treatment of POMS. Registry data from Sweden indicate that between 2011 and 2019, 354 POMS patients were already being treated primarily with highly active substances (77.4% initially and an additional 10.73% after switching), resulting in freedom from disease activity in 83.6% of cases. The median EDSS was 1.75 (IQR 1–2.5) [7]. 

Despite reporting low EDSS scores (with 14 individuals scoring 1, seven scoring 2, and only two scoring 3 or 4), only 11 patients reported no neurological difficulties. The most common symptoms among the remaining 14 were concentration and memory issues, coordination problems, and depressed mood, with corresponding EDSS scores ranging from 1 to 4. It is noteworthy that the patients that reported problems in four or more domains had nevertheless low EDSS scores (1 or 2). This example demonstrates the potential limitations of the EDSS in patient-reported surveys. There may be a lack of awareness among patients about their latest EDSS score or the use of different EDSS versions. Furthermore, the EDSS is not without its own limitations. It is the gold standard for measuring disability, but it requires an in-person assessment and may exhibit high inter- and intra-rater variability, especially at the lower disability levels [12]. According to the current guidelines, depression and euphoria are documented on the scoring sheet but are not taken into consideration. Fatigue may or may not be included in the scoring system, depending on the guidelines per individual study. Taken together, important aspects that might affect the quality of life severely are only weakly represented in the EDSS [13]. 

### 4.3. Quality of Life 

The quality of life was evaluated using the EQ-5D-5L and the MSIS-29. Additionally, the six items from the General Fatigue Assessment (PedQL Fatigue) were used. 

The EQ-5D-5L demonstrated a clear distinction between patients who reported no (8/23) or mild (10/23) or marked (5/23) reduction in quality of life. Anxiety/depression and pain have the greatest impact on patients’ perceived health status. This aligns with the responses provided by patients regarding the symptoms that had the greatest impact on their daily lives documented in clinical characteristics. McKay demonstrated a similar pattern in his cohort using the EQ-5 3L [7]. The majority of patients in his cohort were not affected, with anxiety and depression and pain being the dominant factors in reducing QoL. At present, there is a notable absence of therapeutic modalities that address these topics. Among our cohort, only two respondents (8%) had undergone psychological or neurocognitive therapy, whereas 25% (88/24) had received physiotherapy. This prompts the question of whether active management of anxiety, depression, and pain should be a novel priority, given the considerable impact on quality of life.

The median EQ-VAS, a proxy measure of subjective health perception, was 90, with an interquartile range (IQR) of 78.75 to 96.25, which demonstrated no reduction in comparison to the general German adult population. The normative values for the German population were published with a median of 90 (IQR 80—not calculated) for adults below the age of 40 [14]. However, the response rate for this question in our cohort was only 20 respondents, which might have introduced a bias in the perception of the high quality of life. The only study to date to have been published using EQ-VAS, which was conducted in Sweden, showed a lower EQ-VAS of 75 (interquartile range 60–88.5) based on 345 participants with POMS [7]. 

The MSIS-29 is a scale that indicates a reduction in quality of life, with 0 representing no perceived disability and 100 representing severe disability. The participants in this cohort exhibited a more pronounced yet still slight reduction in quality of life for the MSIS-29 Psychological Score (median 11.1) in comparison to the MSIS-29 Physical Score (median 2.03), which is consistent with the EQ-5D 5L results observed in this cohort. In summary the patients show almost no reduction on QoL using the MSIS-29. The published cohort of McKay had slightly lower QoL measured by MSIS-29 (psychological median 25.00, physiological median 11.25). 

Quality of life (QoL) can be measured with a variety of validated instruments, each addressing QoL in a slightly different manner, which often results in data that cannot be easily compared between each other. For example, a cohort of 51 subjects with POMS and juvenile MS onset before the age of 25 was evaluated using the Paediatric Quality of Life Inventory (PedsQoL) Version 4.0 [15]. The mean total PedsQL score was 68.45 (standard deviation ±18.43; with a value of 100 representing perfect health), indicating a slight reduction in comparison a paediatric population mean of 79.62 (standard deviation ±12.26) [16]. 

Fatigue is another significant factor influencing QoL in MS, regardless of the age at which MS was diagnosed. Two different methods were employed to ascertain whether patients exhibited fatigue. 

The patients were asked to confirm whether they experience fatigue symptoms in their daily lives, which 16% (4/25) confirmed. In contrast, 40.7% of respondents indicated that they had experienced fatigue at least occasionally, as measured by six items from the PedQL general fatigue scale. It appears that respondents may not have considered all aspects of fatigue when responding to the single question inquiring about the presence of fatigue. Thus, it seems mandatory to use structured fatigue questionnaires in order to not underestimated the extend of fatigue. 

## 5. Limitations of the Survey

Given the restricted number of respondents in this feasibility study, further statistical analysis of the findings was not deemed appropriate. A future German-wide project is planned, the objective of which is to incorporate a greater number of potential individuals and to validate the findings. 

The EDSS assessment was not conducted by trained examiners due to the anonymised nature of the survey. It is notable that the most recent EDSS rating provided by patients did not always align with the observed level of disability, which was assessed through the administration of structured questions pertaining to difficulties in various neurological domains (motor, coordination, brainstem, sensory, visual, cognitive, fatigue and depression). Additionally, it is important to acknowledge the inherent limitations of the EDSS. The EDSS is the gold standard for measuring disability, but it requires an in-person assessment and may exhibit high inter- and intra-rater variability, especially at the lower disability levels. Consequently, EDSS scores not evaluated at the time of data collection pose the risk of being inaccurate.

The pilot project assessed the long-term status of patients with POMS who were transitioning into adulthood using an anonymous online survey. Participants were permitted to answer part of the questionnaire; no question was compulsory to complete the survey, so the majority of sections had answers from less than all respondents, which complicated the interpretation of results. For planned future regular repetitions of this survey questions for each section will be mandatory if necessary. Questions on gender will stay optional. 

## 6. Conclusions/Future Directions

The pilot project was designed to assess the feasibility of an online survey tool for obtaining long-term follow-up data on patients with POMS. Even with a single-centre study, new insights into this rare condition could be gathered. The impact of the disease on everyday life was found to be lower than that observed in historical cohorts. The patients were well educated and exhibited a comparatively low burden of neurological symptoms. Conversely, anxiety and depression had a significant impact on perceived health status. These symptoms were not adequately addressed in the majority of affected patients.

These results raise another set of questions that could not be answered given the limited nature of this survey: what are the priorities that should be given to the different symptoms when treating children and adolescents with MS? Is the impact of the aspects of the condition that go beyond the mere presence or absence of focal neurological symptoms well addressed in today’s management of these patients?

To facilitate a more accurate comparison with other centres and countries in the future, it is necessary to determine a minimal data set and a subset of questionnaire for POMS patients transitioning to adulthood.

## Figures and Tables

**Table 1:** Baseline statistics of all participants. Categorial items are presented by the counts of the different options, and numerical items are presented using the median and the interquantile range.

Category	Values	Result
age	Years (mean; SD)	23.66 ± 3.85
sex	female	17
	male	6
	diverse	1
	not disclosed	3
MS type	relapsing remitting	22
	secondary progressive	0
	no answer	5
EDSS	1	14
	2	7
	3	1
	4	1
	Not known	4
MS specific treatment at time of survey	Yes	17
	No	6
	no answer	4
school education	higher education	21
	primary education (completed 8 years)	4
	not completed education	0
	no answer	4
EQ-VAS	Median	90
	Interquantile range	78.75–96.25
	no answer	4
MSIS-29 Physical Score 0–100	Median	2.03
	Interquantile range	0–12.5
MSIS-29 Psychological Score 0–100	Median	11.11
	Interquantile range	2.77–16.67
Fatigue 6 item general fatigue section of PedsQL	Never/rare (%)	59
	Sometimes (%)	18
	Often/almost always (%)	22.5
	no answer	5 participants

**Table 2:** Education levels of the patients.

Educational Level	Number of Patients
higher education	21
primary education (completed 8 years)	4
not completed education	0
no answer	4

**Table 3:** Number of relapses.

Number of Relapses	Number of Patients (Percent)
0	8 (36%)
1	5 (23%)
≥2	9 (41%)

## Data Availability

The original contributions presented in the study are included in the article/Supplementary Materials, further inquiries can be directed to the corresponding author.

## Supplementary Materials

The following supporting information can be downloaded at: https://www.mdpi.com/article/10.3390/children11081024/s1, Figure S1: degree of disability. Number of patients with a certificate of disability and the degree of disability according to the German guidelines Table S1: Reasons for switching DMTs. All reported switching events are listed Table S2: MSIS-29; german questions; Table S3: Items for MSIS-20 Physiological; Table S4: Items for MSIS-29 Psychological; Table S5: Fatigue.

## Glossary

Expanded disability Status Scale (EDSS); multiple sclerosis (MS); paediatric onset multiple sclerosis (POMS); quality of life (QoL).
