# Supplementary material for: Long-Term Socioeconomic and Neurologic Outcome for Individuals with Childhood-Onset Multiple Sclerosis
Source: Children (Basel). 2024 Aug 21;11(8):1024. doi: 10.3390/children11081024 (PMC11352636; doi:10.3390/children11081024)
Supplement: Supplementary file 1 [file children-11-01024-s001.zip › children-3144671-supplementary.pdf]

Supplemental data

Figure S1: degree of disability. Number of patients with a certificate of disability and the degree of disability according to the German guidelines.

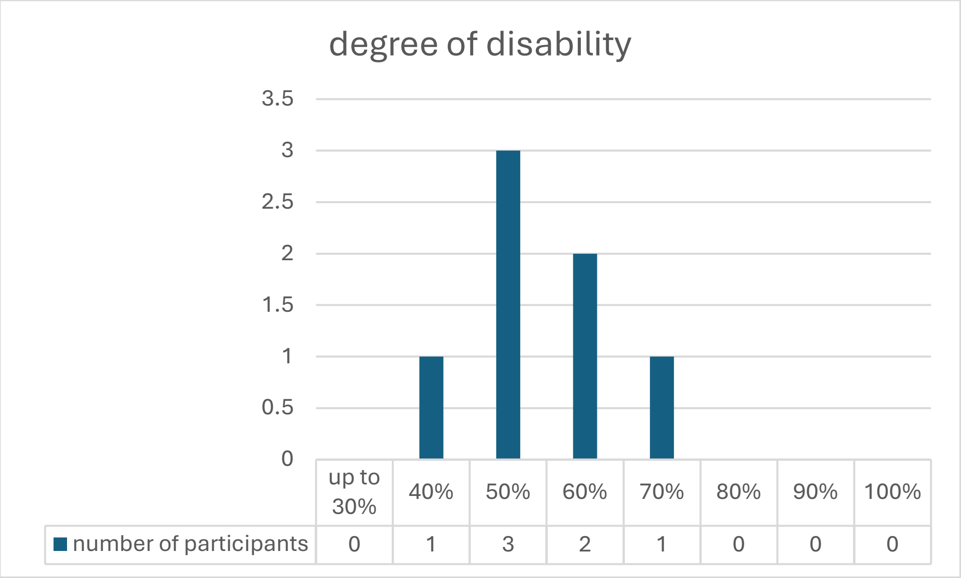

Table S 1 : Reasons for all switching DMTs. **A**ll reported switing events are listed

		reason for switching			
**drug**	**number of respondents taking the drug at any time**	**side effects**	**relapse**	**MRI activity**	**antibodies**
glatiramer acetate	3	3	1	1	0
interferon	10	5	6	0	0
dimethyl fumarate	7	7	1	0	0
teriflunomide	1	0	1	0	0
fingolimod	3	1	2	1	0
natalizumab	3	3	0	0	0
rituximab	1	1	0	0	0

Legend

Table S2 MSIS-29. German questions

Wie schwer fiel es Ihnen in den letzen zwei Wochen ...

1. körperlich anstrengende Dinge zu tun?
2. Dinge fest anzufassen (z.B. Hahn aufdrehen)?
3. Dinge zu tragen?

Hatten Sie in den letzten zwei Wochen…

4. Probleme mit dem Gleichgewicht?
5. Schwierigkeiten, sich in der Wohnung zu bewegen?
6. das Gefühl ungeschickt zu sein?

7. ein Steifigkeitsgefühl?
8. schwere Arme und/oder Beine?
9. Zittern der Arme oder Beine?
10. Krämpfe der Extremitäten?
11. das Gefühl, dass ihr Körper nicht tat, was sie wollten?
12. Beeinträchtigung im sozialen und Freizeitleben zu Hause?
13. Probleme mit den Händen bei Alltagstätigkeiten?
14. Probleme sich fortzubewegen (Auto, Bus, Taxis, Zug)?
15. länger gebraucht, Dinge zu tun?
16. Schwierigkeiten, Dinge spontan zu machen?
17. das Gefühl, ganz schnell zur Toilette zu müssen?

Waren Sie in den letzten zwei Wochen…

18. davon abhängig, dass andere Dinge für sie erledigten?
19. gezwungen, zu Hause zu bleiben?
20. gezwungen die Zeit für Arbeit oder Alltagsaktivitäten einzuschränken?

21. sich allgemein unwohl gefühlt?
22. Schlafprobleme?
23. sich geistig/mental müde gefühlt?
24. Sorgen bezogen auf ihre MS?
25. sich angespannt und ängstlich gefühlt?
26. sich ungeduldig und aufbrausend gefühlt?
27. Konzentrationsprobleme
28. keine Zuversicht?
29. sich traurig/depressiv gefühlt?

Table S3: Items for MSIS-29 physiological . Individual answers to each Question (Q). Each line represents a respondent.

Q1	Q2	Q3	Q4	Q5	Q6	Q7	Q8	Q9	Q10	Q11	Q12	Q13	Q14	Q15	Q16	Q17	Q18	Q19	Q20	raw sum	Score transfor. 0-100
2	1	2	1	1	1	1	1	1	2	1	1	1	1	1	1	1	1	1	1	23	3,75
1	1	1	1	1	1	1	1	1	1	1	1	1	1	2	1	1	1	1	1	21	1,25
1	1		2	1	1	1	1	2	1	1	1	1	1	1	1	1	1	1	1	21	1,25
1	1	1	1	2	1	1	1	1	1	1	1	1	1	1	1	1	**1,05**	**1,05**	**1,05**	21,15	1,4375
1	1	1	2	1	1	1	1	**1,1**	1	1	1	1	1	1	2	1	1	1	1	22,1	2,625
2	1	2	2	2	2	2	1	1	1	2	1	1	1	2	2	2	1	1	1	30	12,5
1	1	1	1	1	1	1	1	1	1	1	1	1	1	1	1	1	1	1	1	20	0
2	1	2	2	1	2	2	3	1	1	1	2	2	1	2	2	2	2	1	1	33	16,25
1	1	1	1	1	1	1	1	1	1	1	1	1	1	1	1	5	1	1	1	24	5
1	**1**	**1**	**1**	1	1	1	1	**1**	**1**	**1**	1	1	1	1	1	1	1	1	1	20	0
3	2	3	5	1	1	1	**2,4**	2		1	5	1	**2,4**	4	4	4	1	3	3	48,8	36
5	1	3	2	1	5	1	3	3	2	2	3	1	1	5	4	1	1	1	1	46	32,5
5	2	2	2	1	3	2	4	2	2	3	3	3	**2,8**	4	3	4	3	3	2	55,8	44,75
3	1	1	1	1	1	1	3	3	1	3	1	1	1	1	1	3	1	1	1	30	12,5
1	1	1	1	1	1	1	1	1	1	1	1	1	1	1	1	1	1	1	1	20	0
1	1	1	1	1	1	1	1	1	1	1	1	1	1	1	1	1	1	1	1	20	0
1	1	1	1	1	1	1	1	1	1	1	1	1	1	1	1	1	1	1	1	20	0
3	1	2	2	1	3	1	4	3	2	2	1	2	1	2	1	3	1	1	1	37	21,25
1	1	1	1	1	1	1	1	1	1	1	1	1	1	1	1	1	1	1	1	20	0
1	1	1	1	1	1	1	1	1	1	2	1	1	1	1	1	1	1	1	1	21	1,25
2	2	1	1	1	1	1	1	1	1	1	1	1	1	2	1	1	1	1	1	23	3,75
1	1	1	1	1	1	1	1	1	1	1	1	1	1	1	1	1	1	1	1	20	0
																		**Sum of all scores**			**196,06**
																		**Median**			**2,03**
																		**Minimum**			**0**

																		**First Quantile**	**0**
																		**Third Quantile**	**12,50**
																		**Maximum**	**44,75**

Table S4: Items for MSIS-29 psychological Individual answers to each Question (Q). Each line represents a respondent.

**Q21**	**Q22**	**Q23**	**Q24**	**Q25**	**Q26**	**Q27**	**Q28**	**Q29**	**raw sum**	**Score transfor. 0-100**
1	1	3	2	1	2	2	1	1	14	13,8)
1	1	3	1	2	1	1	1	1	12	8,34
1	2	2	1	2	1	1	1	2	13	11,11
1	2	1	1	1	1	1	1	1	10	2,78
2	2	1	2	1	1	1	1	1	12	8,33
1	1	1	1	1	1	1	1	1	9	0
2	3	2	3	2	4	5	1	2	24	41,67
1	1	1	1	1	1	1	1	1	9	0
1	**1**	1	1	1	1	1	**1**	1	9	0
3	5	5	5	3	2	4	1	2	30	58,33
5	4	5	1	5	5	4	5	5	39	83,33
4	3	5	3	2	4	4	4	3	32	63,89
**1,1**	1	1	2	1	1	1	1	1	10,1	3,056
2	2	2	2	1	1	1	1	2	14	13,89
1	2	1	1	1	1	1	1	1	10	2,78
1	2	1	1	1	1	1	1	1	10	2,78
3	4	5	3	5	3	4	5	5	37	77,78
1	1	1	2	3	1	2	2	2	15	16,67
1	1	1	1	1	1	1	1	1	9	0
2	2	2	2	2	1	1	1	1	14	13,89
1	1	2	1	2	2	1	1	2	13	11,11
							**Sum of all scores**			**433,61**
							**Median**			**2,78**
							**Minimum**			**0**
							**First Quantile**			**11,11**

							**Third Quantile**	**16,67**
							**Maximum**	**83,33**

Table S5: Fatigue. Individual answers to each Question (Q). Each line represents a respondent. Transformation to a score of 0-100 was done according to [2]. Missing data was added in accordance to published guidelines [2].

Questions

Q2	**Ich fühle mich körperlich schwach**
Q3	**Ich fühle mich zu müde, um Dinge zu tun, die ich mag.**
Q4	**Ich fühle mich zu müde, um Zeit mit Freunden zu verbringen.**
Q5	**Ich habe Schwierigkeiten, etwas, das ich angefangen habe, zu Ende zu bringen.**
Q6	**Ich habe Schwierigkeiten, etwas zu beginnen.**

raw data according to Lickert scale							
**Q1**	**Q2**	**Q3**	**Q4**	**Q5**	**Q6**		
2	2	0	0	2	2		
2	0	0	0	0	2		
1	0	0	1	0	0		
0	0	0	0	0	0		
1	0	0	1	0	0		
0	1	1	1	0	0		
0	0	0	0	0	0		
4	2	2	3	3	2		
2	0	0	0	0	0		
2	2	2	1	1	0		
4	3	3	4	1	1		

4	4	3	3	0	0		
2	3	4	3	4	3		
2	2	1	0	0	0		
3	1	2	1	1	1		
3	0	0	0	0	0		
3	0	0	0	0	0		
3	1	3	2	2	3		
1	0	0	0	0	1		
1	1	0	0	0	0		
2	1	1	1	0	0		
2	2	1	1	1	1		
**Data shown after linear transformation 0 -100**							
**Q1**	**Q2**	**Q3**	**Q4**	**Q5**	**Q6**	**Sum**	**Individual value**
50	50	100	100	50	50	400	66,6666667
50	100	100	100	100	50	500	83,3333333
75	100	100	75	100	100	550	91,6666667
100	100	100	100	100	100	600	100
75	100	100	75	100	100	550	91,6666667
100	75	75	75	100	100	525	87,5
100	100	100	100	100	100	600	100
75	50	50	25	25	50	275	45,8333333
50	100	100	100	100	100	550	91,6666667
50	50	50	75	75	100	400	66,6666667
0	25	25	0	75	75	200	33,3333333
0	0	25	25	100	100	250	41,6666667
50	25	0	25	0	25	125	20,8333333

50	50	75	100	100	100	475	79,1666667
25	75	50	75	75	75	375	62,5
25	100	100	100	100	100	525	87,5
25	100	100	100	100	100	525	87,5
25	75	25	50	50	25	250	41,6666667
75	100	100	100	100	75	550	91,6666667
75	75	100	100	100	100	550	91,6666667
50	75	75	75	100	100	475	79,1666667
50	50	75	75	75	75	400	66,6666667
					**Mean ± SD**	**73,11 ± 23,14**	
					**Median**	**81,25**	
					**5% (eq. to -2SD)**	**33,75**	
					**Min**	**20,83**	
					**32% (eq. to -1SD)**	**66,67**	
					**First Quantile**	**63,54**	
					**68% (eq to +1SD)**	**88,67**	
					**Third Quantile**	**91,67**	
					**95% (eq to +2SD)**	**99,58**	
					**Max**	**100**	
